# *AMY1* gene copy number associated with xerostomia and Sjögren’s syndrome: a cross-sectional study

**DOI:** 10.1186/s12903-025-05595-1

**Published:** 2025-02-14

**Authors:** Indre Stankeviciene, Alina Puriene, Vilma Brukiene, Diana Mieliauskaite, Synnøve Bække, Berit Tommeras, Rania Al-Mahdi, Arunas Rimkevicius, Lina Stangvaltaite-Mouhat

**Affiliations:** 1 Institute of Dentistry, Faculty of Medicine, Vilnius University, Vilnius, 01131 Lithuania; 2 Department of Experimental, Preventive and Clinical Medicine, State Research Institute Center for Innovative Medicine, Vilnius, Lithuania; 3 UiT The Arctic University of Norway, Tromso, Norway; 4 Oral Health Centre of Expertise in Eastern Norway, Oslo, Norway

**Keywords:** Adults, Salivary α-amylase, Gene copy number, Genetics, Dry mouth, Xerostomia, Sjögren’s syndrome

## Abstract

**Background:**

Dry mouth-related conditions adversely affect patients’ well-being, as well as their oral and general health. There are indications that the quantitative and qualitative protein composition of saliva is disrupted in patients with xerostomia and Sjögren’s syndrome. Salivary α-amylase levels positively correlate with the copy number (CN) of its coding gene, *AMY1* (amylase alpha 1). This study aimed to investigate the association between intensity of xerostomia, Sjögren’s syndrome, and *AMY1* CN. Establishing such an association could position *AMY1* CN as a potential genetic biomarker for dry mouth-related conditions, aiding in their early detection.

**Methods:**

This cross-sectional study utilized secondary data from the main dry mouth study conducted in five public hospitals in Vilnius, the capital city of Lithuania. Based on sample size calculations for the main study, 127 patients who met the inclusion criterion of dry mouth complaints (sometimes, often, and always) were recruited. The outcomes were xerostomia intensity, assessed using a visual analogue scale (VAS), and confirmed cases of Sjögren’s syndrome, assessed using the 2016 ACR/EULAR classification criteria and classified as either primary or secondary. Sociodemographic information included age and sex; self-perceived stress levels were assessed using the Perceived Stress Scale (PSS-10). During clinical examinations, unstimulated whole sialometry was performed for 15 min, and oral mucosa swabs were collected. The swabs were used to quantify *AMY1* CN via droplet digital PCR (ddPCR). Data were analyzed using both univariable and multivariable regression models.

**Results:**

In total, 112 patients with available *AMY1* CN data and recorded xerostomia intensity VAS scores were included in this study. Of these, 26 (23%) were diagnosed with Sjögren’s syndrome; 9 (8%) had primary and 17 (15%) had secondary Sjögren’s syndrome. According to multivariable linear regression analyses, higher *AMY1* CN was associated with 0.15 lower xerostomia intensity VAS score (β =-0.15, 95% CI -0.30, -0.01). Higher *AMY1* CN reduced the odds for primary Sjögren’s syndrome (OR 0.52, 95% CI 0.03–0.89).

**Conclusions:**

The present study indicated an inverse association between xerostomia, primary Sjögren’s syndrome, and *AMY1* CN. Studies validating these findings and exploring the underlying mechanisms are warranted.

## Background

Dry mouth is an umbrella term covering several different conditions. Xerostomia is a subjective feeling of mouth dryness, and hyposalivation is a condition of objective reduction in saliva volume. Individuals may experience these conditions separately or simultaneously [1, 2]. Both subjective and objective mouth dryness were identified in 11–15% of cases [3]. Multiple conditions can cause dry mouth, for example, chronic systemic diseases, use of medication, radiotherapy in the neck and head region, or elevated stress levels [4–6]. Sjögren’s syndrome is an autoimmune condition that is known for its direct effect on exocrine salivary glands, described as lymphocytic infiltration of its epithelium, which often results in both xerostomia and hyposalivation [7, 8]. Dry mouth, despite its type or etiology, is related to lower quality of life, sleep disturbances, difficulty in speaking, and an increased risk for general and oral diseases, especially dental caries and candidiasis [2, 4, 7]. In addition, patients with Sjögren’s syndrome may experience various systemic pathologies and have a higher risk for non-Hodgkin lymphoma [8]. Therefore, early detection of dry mouth-related conditions is important.

The severity of xerostomia may not be related to the severity of hyposalivation, i.e., the amount of saliva produced by salivary glands [9, 10]. In addition, in Sjögren’s syndrome cases, the saliva volume does not correlate to the level of salivary gland damage. It has been suggested that in cases of xerostomia and Sjögren’s syndrome, there is a disbalance in quantitative and qualitative protein composition in saliva [10]. A literature review published in 2022 found an association between both hyposalivation and xerostomia and decreased levels of MUC5B salivary protein. This reduction may disrupt essential functions of saliva, such as water retention, lubrication, and viscoelastic properties. However, the authors also emphasized that other proteins could play significant roles in these processes [11]. Another study identified 15 proteins with differential expression levels, including α-amylase precursors, in individuals with primary Sjögren’s syndrome compared to healthy controls and those with other forms of dry mouth [12]. There are indications that α-amylase may be involved in inflammatory mechanisms in Sjögren’s syndrome development, as it was observed to serve as a salivary gland-specific T cell epitope resulting in the initiation of autoimmunity [13]. The salivary α-amylase is the most abundant salivary protein. This protein participates in digestion, fermenting starch into smaller saccharides [10]. Salivary α-amylase is encoded by the gene amylase alpha 1 (*AMY1*), and due to evolutionary development related to increased carbohydrate consumption, each person has between 2 and 16 copies of this gene. It has been demonstrated that the levels of α-amylase in saliva were positively correlated with *AMY1* copy number (CN) [14–17]. The aim of the present study was to investigate the possible association between the intensity of xerostomia, Sjögren’s syndrome, and *AMY1* CN. If such an association is established, it highlights *AMY1* CN potential to serve as a genetic biomarker of dry mouth-related conditions, facilitating early detection.

## Methods

### Study design and settings

This cross-sectional study was based on secondary data analysis, which was initially collected for the main dry mouth study in five public hospitals in Vilnius, the capital city of Lithuania. Data was collected between 2020 and 2022.

### Participants and sample size

A sample size of 126 participants was calculated for the main dry mouth study. For this, the calculation was performed using the G-Power calculator based on β = 95% and α = 5%. In total, 127 patients who met the inclusion criterion of experiencing dry mouth (sometimes, often, and always) were recruited. *AMY1* CN could be retrieved for 113 (89%) participants; out of them, one did not provide data for xerostomia intensity. Consequently, data from 112 participants were included in this study.

### Variables

Xerostomia intensity, measured using a Visual analogue scale (VAS), was an outcome of this study. The participants were invited to indicate how intense were their xerostomia symptoms past week on a scale from 0 to 10, where 0 meant ‘no symptoms’ and 10 ‘very intense symptoms’ [18].

The other outcome was Sjögren’s syndrome (confirmed by a rheumatologist according to 2016 ACR/EULAR criteria ‘yes’ versus ‘no’), the detailed description of the confirmation process of Sjögren’s syndrome can be found elsewhere [19]. Participants with confirmed condition were further classified into primary Sjögren’s syndrome and secondary Sjögren’s syndrome groups. Primary Sjögren’s syndrome referred to cases occurring in individuals without other autoimmune conditions, while secondary Sjögren’s syndrome was diagnosed in those with coexisting autoimmune disorders [20].

Sociodemographic determinants (age and sex) were collected using the World Health Organization (WHO) Oral Health Questionnaire for Adults and self-perceived stress levels were measured using the Perceived Stress Scale-10 (PSS-10). The questionnaires were translated from the English language to Lithuanian language, and back-translated by two independent persons; the minor inconsistencies were corrected. Then the questionnaire was pilot-tested in 10 adults. The Lithuanian version of the questionnaire was previously used in the National Oral Health Survey [19].

Unstimulated whole sialometry (UWS) and oral mucosa swabs were collected during clinical examination, which was performed according to the WHO Oral Health Survey Basic Methods recommendations and the procedure described in detail elsewhere [19]. The unstimulated salivary flow was collected for 15 min in a graded tube (Falcon^®^, UK) in a calm environment with patients sitting. The participants were instructed not to eat, drink, brush their teeth or smoke at least one hour before the test.

Buccal mucosa swabs (hDNAfreeFLOQSwabs^®^, Italy) were stored at a temperature of − 20 °C and then transported to UiT The Arctic University of Norway, Norway for genetic analysis.

### Genetic analyses

#### DNA extraction from oral mucosa swabs

The DNA was extracted manually from the oral mucosa swab samples using the QIAamp DNA Mini Kit (Qiagen, Heidelberg, Germany) according to the buccal swabs protocol. Agarose gel electrophoresis was performed to check the quality and yield of the isolated DNA and the concentration of the DNA was measured using the Qubit 3.0 fluorometer (Life Technologies CA, USA) according to the manufacturer’s instructions.

#### ddPCR for gene copy number determination

The QX200TM Droplet Digital TM PCR system (Bio-Rad, Pleasanton, CA) was used in the current study to determine the CN of the *AMY1* gene from the extracted DNA. A total volume of 22 µl of the reaction mixture was transferred into the sample well of the cartridge, and 70 µl of droplet generation oil was applied to the correspondent oil well prior to placing the gasket over the cartridge and transferring it into the droplet generator. Primers, probes, and restriction enzyme used were described previously [21]. After droplet generation, 40 µl of the sample emulsion was transferred into a 96-well PCR plate (Eppendorf, Germany) and then heat sealed with a pierceable foil using the PX1™ PCR Plate Sealer (Bio-Rad, USA) at 180 °C for 5 s. PCR amplification was performed using a C1000TM Thermal cycler (Bio-Rad). The plate was first incubated at 37 °C for 30 min for enzymatic digestion. The conventional PCR was run at 95 °C for 10 min, 40 cycles of 95 °C for 30 s and 58 °C for 1 min, and 98 °C for 10 min. After PCR, the droplets were analysed by the Bio-Rad QX200 Droplet Reader. Samples known to have 6 and 14 diploid copies of *the AMY1 gene (NA18972*,* NA18956 respectively*,* NHGRI Sample Repository for Human Genetic Research*,* Coriell Institute for Medical Research*,* Camden*,* NJ*,* USA)* were included as positive control and DNase-free water was used as a negative control.

The generated data to determine the copy number of *AMY1* were analyzed using the QuantaSoft software version 1.7.4.0971 (Bio-Rad). The threshold to distinguish positive droplets from negative ones was set for each reaction automatically by the software if not stated otherwise. If needed, further analysis of the data was done using the QuantaSoftTM PRO software (version 1.0).

### Statistics

The SPSS version 27 (IBM, Armonk, NY, USA) was used for statistical analyses.

Mean, standard deviation (SD) and median and interquartile range (IQR) were calculated for continuous variables and frequencies for categorical variables in Table 1.

Based on skewness and kurtosis, the xerostomia VAS data was normally distributed [22]; therefore, we employed univariable and multivariable linear regression analyses to examine the association between *AMY1* CN and xerostomia in Table 2. R^2^, which shows how much variation in the outcome is explained by independent variables, was recorded.

Univariable and multivariable binary logistic regression analyses were used to investigate an association between *AMY1* CN and primary and secondary Sjögren’s syndrome in Table 2. Nagelkerke R^2^, which is somewhat similar to R^2^ in linear regression, was recorded.

Based on Tolerance and VIF values, there were no indications of multicollinearity. The level of significance was set at *p* < 0.05, β coefficients and odds ratios (OR) are presented with their 95% confidence intervals (CI).

## Results

### Characteristics of participants

The study sample consisted of 94 (84%) females, and the mean age of the participants was 64 (standard deviation (SD) 17.25)) years (Table 1). Mean xerostomia VAS score was 5.88 (SD 2.14). One participant indicated a VAS score of 0 and three participants– a VAS score of 10. Among the 112 participants with xerostomia, 26 (23%) were diagnosed with Sjögren’s syndrome, including 9 (8%) with primary and 17 (15%) with secondary Sjögren’s syndrome.

**Table 1:** Characteristics of all 112 (100%) study participants having xerostomia. Out of them, primary Sjögren’s syndrome was diagnosed in 9(8%), and secondary Sjögren’s syndrome in 17 (15%) of the participants

Characteristics	Xerostomia *n* = 112 (100%)	Primary Sjögren’s syndrome *n* = 9 (8%)	Secondary Sjögren’s syndrome *n* = 17 (15%)
Xerostomia VAS Mean (SD)			
	5.88 (2.14)	6.56 (1.81)	5.59 (2.55)
*AMY1* CN Median (IQR)			
	6.00 (3.00)	4.00 (1.50)	7.00 (4.50)
Age, years Mean (SD)			
	64.17 (17.25)	53.22 (13.75)	56.35 (8.14)
Sex, n (%)			
*Female*	94 (84%)	8 (89%)	16 (94%)
*Male*	18 (16%)	1 (11%)	1 (6%)
UWS Mean (SD)			
	2.01 (1.87)	1.81 (0.96)	2.81 (1.78)
PSS-10 Mean (SD)			
	18.73 (7.86)	21.56 (5.77)	19.12 (8.53)

CN– copy number, IQR– interquartile range, PSS-10– perceived stress scale-10, SD– standard deviation, UWS– unstimulated whole sialometry ml/15 min, VAS– visual analogue scale

### Association between intensity of xerostomia, Sjögren’s syndrome and AMY1 CN

According to multivariable regression analysis, higher *AMY1* CN associated with 0.15 lower xerostomia VAS score (β =-0.15, 95% CI -0.30, -0.01) (Table 2). Higher *AMY1* CN reduced odds for primary Sjögren’s syndrome (versus not diagnosed Sjögren’s syndrome) (OR 0.52, 95% CI 0.03–0.89). Additionally, UWS was independently inversely associated with xerostomia VAS score (β = -0.22, 95% CI -0.44, -0.01).

**Table 2:** Unstandardized β coefficients and their 95% confidence intervals (CI) for the association between the intensity of xerostomia and *AMY1* copy number according to univariable and multivariable linear regression analyses. Odds ratios (OR) and their 95% confidence intervals for the association between primary and secondary Sjögren’s syndrome (versus not diagnosed Sjögren’s syndrome) as outcomes and *AMY1* copy number according to univariable and multivariable binary logistic regression analyses

	Xerostomia VAS		Sjögren’s syndrome			
Variables	Crude β 95% CI	Adjusted β 95% CI	Primary Crude OR 95% CI	Primary Adjusted OR 95% CI	Secondary Crude OR 95% CI	Secondary Adjusted OR 95% CI
*AMY1* copy number (continuous)	-0.16 (-0.30, -0.01)	-0.15 (-0.30, -0.01)	0.52 (0.31–0.88)	0.52 (0.30–0.89)	1.05 (0.88- 1.26)	1.07 (0.88- 1.30)
Age, year (continuous)		0.01 (-0.01, 0.04)		0.96 (0.92–0.99)		0.97 (0.94- 1.00)
Sex *Female (ref.)*		0.50 (-0.61, 1.60)		1.00 (0.09–11.15)		4.58 (0.48–44.12)
*Male*						
UWS, ml/15 min (continuous)		-0.22 (-0.44, -0.01)		0.84 (0.46–1.56)		1.26 (0.97–1.64)
PSS-10 (continuous)		0.03 (-0.03, 0.08)		1.02 (0.92–1.14)		0.99 (0.92–1.06)
R^2^ for linear regression/ Nagelkerke R^2^ for logistic regression		0.108		0.293		0.158

PSS-10– perceived stress scale-10, UWS– unstimulated whole sialometry ml/15 min, VAS– visual analogue scale

## Discussion

To the best of our knowledge, this is the first study to indicate an inverse association between intensity of xerostomia, primary Sjögren’s syndrome and *AMY1* CN.

Our study showed that in participants suffering from xerostomia one *AMY1* CN increment resulted in a 0.15 score lower xerostomia intensity VAS. The evidence explaining this relationship remains limited. Previous studies have demonstrated that salivary α-amylase activity and levels positively correlate with *AMY1* CN [14–17]. Furthermore, there are indications that the regulation of salivary α-amylase secretion may also be closely linked to salivary flow rate [14, 23–24]. A study in diabetic rats investigating changes in salivary properties after treatment with Ixeris dentata, a natural compound believed to regulate the salivary α-amylase secretion mechanism, found increases in both salivary α-amylase levels and salivary flow rate [25]. In the present study, salivary flow rate was included in the model and was found to be independently and inversely associated with xerostomia intensity, as measured by the VAS score. One of the potential mechanisms between *AMY 1* CN and xerostomia may be that higher *AMY1* CN results in higher α-amylase levels in saliva, leading to lower dry mouth feeling.

Another potential mechanism linking *AMY1* CN to xerostomia could involve insulin resistance. Studies have shown that *AMY1* CN is lower in overweight and obese individuals with insulin resistance, who are also at higher risk for cardiovascular disease and inflammatory processes [26]. It is possible that lower *AMY1* CN is associated with the onset of chronic systemic diseases, with xerostomia emerging as a consequence of these conditions. Additionally, there is evidence that changes in salivary α-amylase levels may disrupt the balance of oral microbiota, potentially resulting from altered carbohydrate processing [27]. Other studies have suggested that individuals with lower *AMY1* CN may pass poorly digested starch further into the digestive tract, providing a substrate for microbial metabolism and thereby influencing microbiota composition [28]. Changes in gut microbiota may contribute to the initiation and progression of chronic systemic diseases [29]. Furthermore, *AMY1* CN appears to be involved not only in carbohydrate metabolism but also in fat metabolism, which has also been linked to the development of diseases such as cardiovascular disease. This suggests a more complex relationship between *AMY1* CN and non-communicable diseases [30–31].

Further research is needed to explore the mechanisms underlying the relationship between *AMY1* CN and xerostomia, including investigations into salivary α-amylase levels. The clinical significance of a 0.15 VAS score difference warrants consideration. A study examining the association between xerostomia VAS and quality-of-life measures found that each unit increase in VAS score was associated with a 0.49-point increase in the OHIP-14 score, indicating that even slight differences in VAS scores may have meaningful implications for individuals [32].

Our study indicated that one additional copy of *AMY1* resulted in 0.48% lower odds for patients with xerostomia as well have the diagnosis of primary Sjögren’s syndrome. The association of *AMY1* CN with Sjögren’s syndrome could be explained by the involvement of this gene in regulating the immune system. Lower *AMY1* CN was linked to increased inflammatory parameters [26]. In another study, this relationship with inflammatory patterns resulted in a positive association between *AMY1* CN and IL-10, which is known to play a role in anti-inflammatory functions and preventing autoimmune disorders [33]. Moreover, this link could be explained not only by changes in immune responses and the secretion of anti-inflammatory factors but also by the role of salivary α-amylase as a potential trigger for autoimmune reactions. A study conducted over 20 years ago hypothesized that salivary α-amylase might initiate the autoimmune process in Sjögren’s syndrome by binding to T cells in the salivary glands and acting as an autoantigen [13]. It is possible that the body begins to recognize its own protein, salivary α-amylase, as an autoantigen when it is secreted at lower levels. This reduced secretion may hinder the immune system from developing sufficient tolerance, thereby allowing the protein to act as a trigger for autoimmunity. Further research is needed to validate and elucidate these findings.

This study was based on a secondary analysis, which may be interpreted as a limitation. On the other hand, using already collected data saves resources and minimizes patient burden as there is no need for additional recruitment and examination. Consequently, the sample size was not calculated particularly for this study. A larger sample size could reveal more clinically significant results, as in this study, an increase of one *AMY1* CN resulted in a 0.15 higher xerostomia intensity VAS score. Moreover, there were only nine patients with primary Sjögren’s syndrome, but the crude and adjusted associations between primary Sjögren’s syndrome and *AMY1* CN were statistically significant. Another limitation is that the study utilized a convenience sample. To mitigate this, we employed a clear inclusion criterion. Additionally, information bias cannot be ruled out, as xerostomia intensity was assessed using a subjective self-reported instrument inquiring to report symptoms during the past week. Participants may have had different perceptions of their symptoms, and the intensity of xerostomia can fluctuate over time, making it difficult to obtain a stable measurement. To address this, we utilized the validated VAS tool, which is recognized as reliable in assessing subjective symptoms. Due to these limitations and the multifactorial origin of these conditions, the findings of the present study should be interpreted with caution.

This study highlights the potential clinical significance of AMY1 CN as a biomarker for xerostomia and primary Sjögren’s syndrome. Identifying individuals with a higher risk based on AMY1 CN could enable clinicians to facilitate earlier diagnoses and implement timely interventions, thereby enhancing patient care and improving clinical outcomes.

## Conclusion

The present study indicated an inverse association between xerostomia, primary Sjögren’s syndrome, and *AMY1* CN. Studies validating these findings and exploring the underlying mechanisms are warranted.

## Data Availability

The data that support the findings of this study are available from the corresponding author (IS, indre.stankeviciene@mf.vu.lt) upon reasonable request.

## Abbreviations

AMY1: Alpha amylase 1
CI: Confidence interval
CN: Copy number
ddPCR: Digital droplet polymerase chain reaction
DNA: Deoxyribonucleic acid
OR: Odds ratio
PPS: 10-perceived-stress scale 10
WHO: World Health Organization
